# A Wearable Force Myography-Based Armband for Recognition of Upper Limb Gestures

**DOI:** 10.3390/s23239357

**Published:** 2023-11-23

**Authors:** Mustafa Ur Rehman, Kamran Shah, Izhar Ul Haq, Sajid Iqbal, Mohamed A. Ismail

**Affiliations:** 1 Department of Mechatronics Engineering, University of Engineering and Technology Peshawar, Peshawar 25000, Pakistan; urr.mustafa@gmail.com (M.U.R.);; 2 Department of Mechanical Engineering, King Faisal University, Al-Ahsa 31982, Saudi Arabia; maismail@kfu.edu.sa; 3 Department of Information Systems, King Faisal University, Al-Ahsa 31982, Saudi Arabia; siqbal@kfu.edu.sa

**Keywords:** force myography, force-sensitive resistor, gesture recognition

## Abstract

Force myography (FMG) represents a promising alternative to surface electromyography (EMG) in the context of controlling bio-robotic hands. In this study, we built upon our prior research by introducing a novel wearable armband based on FMG technology, which integrates force-sensitive resistor (FSR) sensors housed in newly designed casings. We evaluated the sensors’ characteristics, including their load–voltage relationship and signal stability during the execution of gestures over time. Two sensor arrangements were evaluated: arrangement A, featuring sensors spaced at 4.5 cm intervals, and arrangement B, with sensors distributed evenly along the forearm. The data collection involved six participants, including three individuals with trans-radial amputations, who performed nine upper limb gestures. The prediction performance was assessed using support vector machines (SVMs) and k-nearest neighbor (KNN) algorithms for both sensor arrangments. The results revealed that the developed sensor exhibited non-linear behavior, and its sensitivity varied with the applied force. Notably, arrangement B outperformed arrangement A in classifying the nine gestures, with an average accuracy of 95.4 ± 2.1% compared to arrangement A’s 91.3 ± 2.3%. The utilization of the arrangement B armband led to a substantial increase in the average prediction accuracy, demonstrating an improvement of up to 4.5%.

## 1. Introduction

The amputation of an individual’s limb has far-reaching effects on the patient and their family. Beyond the physical challenges of the disability, the limited mobility, and persistent muscle pain, it profoundly influences the patient’s psychological well-being. Studies suggest that psychological disorders are prevalent among amputees, ranging from 32% to 84%, with depression rates reaching as high as 63% and post-traumatic stress disorder affecting approximately 3.3% to 56.3% of individuals in this population [1]. According to the World Health Organization (WHO), 15% of the world’s population suffers from a disability [2]. Among these, limb amputation is one of the significant contributors to this figure, making it a global issue. The major causes of limb amputations include accidents, peripheral vascular diseases, tumors, infections, diabetes, and congenital conditions [3,4]. Public Health England (PHE) reported 27,465 amputations between 2015 and 2018, compared with 24,181 cases between 2012 and 2015—a rise of 14% [5], whereas the National Health Service revealed 17,845 upper limb amputations in Scotland between 1981 and 2013 [6]. Similarly, Ziegler et al. [7] estimated that there are 41,000 upper limb amputees in the United States, and this is expected to double by 2050.

Upper limb amputations can be categorized as trans-radial (below-elbow) and trans-humeral (above-elbow) amputations. Semasinghe et al. [8] reported that trans-radial amputations contribute 60% to the total number of upper limb amputees. Therefore, the robotic community is putting great effort into rehabilitating and creating a barrier-free environment by developing technologically advanced prosthetic devices. These prostheses are either body-powered or externally powered devices enabling amputees to perform the activity of daily livings (ADLs) independently. However, externally powered active prostheses offer more comfort, functionality, and cosmetic restoration than body-powered prostheses.

Although bionic hands can provide most of the functionalities of human hands, they are still not well-recognized at the commercial level. The statistics reveal that 20% of adult amputees reject prosthetic devices. Of those who accept a prosthesis, 26% of adults and 45% of children are dissatisfied with their devices and choose not to wear them. The major reasons for rejection include poor actuation, the absence of intelligent and precise sensor function, a lack of aesthetics, and a heavy weight [9].

Externally powered trans-radial upper limb prostheses are controlled through various inputs and interfaces. Research has been carried out since their development to develop precise, accurate, and efficient signal acquisition techniques for these devices. The most commonly and widely used sensing mechanism in research-based and commercial prostheses is surface Electromyography (sEMG) electrodes [10]. These electrodes measure neuromuscular activity, which can then be used to extract the user’s intent [11]. Various EMG signal-extraction and processing methods can be used for this purpose. Conventional methods involve a measurement of the muscle contraction intensity through these electrodes, to be used as an input to the control system [12]. Although these techniques are widely implemented in commercial products, a few fundamental issues are associated with this approach, including instability and the low amplitude of sEMG electrodes, ranging from a few hundred micro-volts to a few milli-volts [13].

Furthermore, EMG electrodes are sensitive, and even the slightest limb movement may disturb the acquired signal, resulting in a reduced signal-to-noise ratio. Moreover, sEMG electrodes require excellent skin contact for acquiring high-quality myoelectric signals. Also, the calibration of EMG electrodes is often disturbed by hairy or sweaty skin [14]. Other drawbacks of sEMG signals include their variable nature due to sweat, electrode shifts, motion artefacts, and crosstalk among deep adjacent muscles [15,16,17]. Another significant advantage of FMG over EMG is its capability to minimize the electromechanical delay (EMD) [18,19,20,21]. FMG exhibits a slight delay in generating electrical signals from muscle movements compared to raw EMG, given that the initiation of muscle contraction involves the initial electrical activity within the muscle cell. Notably, raw EMG signals necessitate rectification and filtration for effective utilization in human–machine interface control. These processing steps introduce a computational delay in the signal generation. In contrast, FMG has the ability to predict the onset of a muscle contraction without the need for additional processing steps. The pre-processing of EMG signals accumulates time and causes a delay between muscle activity and motion actuation. This delay may result in frustration for amputees as the limb fails to respond to muscle activity in real-time, and it may lead to the rejection of the prosthetic device [22,23].

Besides sEMG, there are other techniques that have been developed, such as ultrasound imaging [24], mechanomyography [25], targeted muscle reinnervation (TMR) [26], and force myography (FMG) [27], to monitor the physical activities of the limbs and convert them into useful information for controlling prostheses. However, FMG has gained increasing attention among the researcher’s community over the last few years. Several prosthetic prototypes have been tested using FMG technology, as documented in references [28,29,30]. This non-invasive method interprets the limb movement or position by analyzing variations in the stiffness of the muscle–tendon complex (MC) relative to its default state. Various sensors, including piezoelectric [31], piezoresistive [25], and capacitive [27] sensors, have been employed to interpret limb muscle position.

Nonetheless, force-sensitive resistors (FSRs) have gained greater popularity and widespread use in this approach. FSR sensors exhibit resistance changes in response to an applied force or pressure. FMG technology was initially introduced by Phillips and Craelius [32] to successfully create pressure topographic maps based on muscle pressure against the socket. Subsequently to their pioneering work, numerous researchers have explored the applications of FMG in trans-radial prostheses. FMG technology has been applied for the detection of upper limb activities, including hand gesture classification [33], finger forces [34], grip strength [35], precise finger motions [36], and the monitoring of wrist and forearm movements [37]. The development of FMG systems lacks a universally accepted standard regarding the number of sensors and the essential sampling rate for efficiently detecting upper limb movements. Several studies [2,38,39] have utilized low-density pressure mapping, typically incorporating three to eight sensors, while others have designed specialized sensor arrays for FMG applications [40,41]. Although high-density sensor arrays have shown more favorable results compared to low-density sensor FMG bands, they introduce additional complexity, weight, costs, and computational demands into the system. This, in turn, leads to increased maintenance and manufacturing expenses for prostheses. As for the sampling rates, there is considerable variation across studies, ranging from 6 Hz to 1 kHz. While a sampling rate of 6 Hz may suffice for recognizing static gestures, dynamic gestures involving a combination of movements, such as those seen in multiple hand, forearm, and elbow actions, benefit from higher sampling frequencies [27].

Many studies [2,34,37,42] have been conducted to perform comparative studies between FMG and other sensing techniques, and it was evaluated that FMG outclasses its performance compared to other available techniques. However, most studies [25,32,43,44,45,46] have utilized multiple FSR sensors embedded within a band/socket to classify multiple grasps for anthropomorphic prostheses. These studies lacked an essential design of casing for FSRs that could be used generically for all the amputees and fitted inside a socket. In addition, healthy subjects were used during the data collection and classification phase because they could effectively produce distinguishable muscle signals for various movements. Also, using multiple sensors may increase the system complexity, cost, and computational time, resulting in increased production and maintenance costs for prostheses that make them beyond the reach of most amputees in developing countries.

This research article presents a novel wearable FMG armband that provides an innovative solution for detecting user intent in upper limb gestures. Expanding on our prior research by focusing on optimizing the sensor quantity and sampling frequency for the armband, this study continued that research seamlessly [47]. The specially designed casing for FSR sensors sets this armband apart from others, allowing for effective and efficient mounting on the user’s forearms. Furthermore, our study examined two sensor arrangements implemented on this newly developed FMG armband. The performance of these arrangements was thoroughly examined through a rigorous assessment involving machine learning classifiers.

## 2. Materials and Methods

This study developed the FMG setup and an experimental protocol for upper extremity gesture recognition.

### 2.1. Development of FMG Band

The developed FMG hardware had two major hardware components. One was FSR casings, and the other was the signal extraction and conditioning setup.

#### 2.1.1. Design of FSR Casing

**Figure 1:**
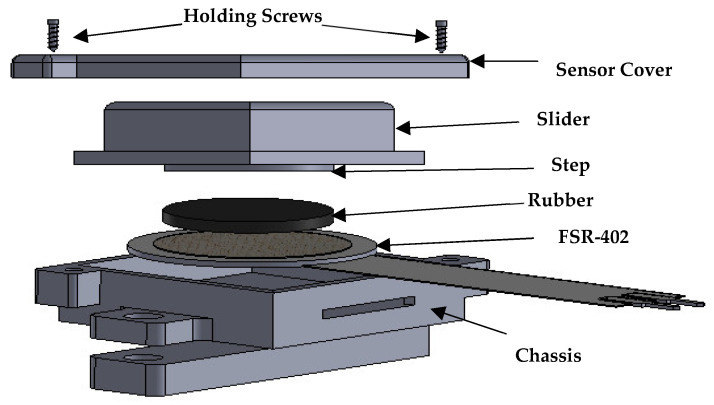
Step-by-step illustration of FSR placement between both casings.

In this study, seven interlinked force-sensitive resistors (FSRs) (model 402) were selected for converting muscle pressure to electric signals. An FSR is a sensitive variable resistor that works on the principle of a decrease in resistance with an increase in applied force/pressure. Attaching FSRs directly to the human body may result in unwanted outputs due to uneven pressure from MC stiffness or the improper bending of the FSR, which eventually leads to damage and/or undesired signals from the FSR [27,48]. Therefore, a customized casing for each FSR sensor was designed to protect the FSR from bending and eliminate these uneven MC pressures. The FSR casing comprised (1) a chassis and (2) a slider. The FSR sensor was placed inside the chassis part of the casing. The slider of the casing was designed to have the primary function of transferring the MC force from the forearm onto the FSR sensor. The upper end of this slider part, having dimensions of 16 × 16 mm, was directly in contact with the subject’s skin, whereas the lower end slide inside the chassis of the casing. A purpose-designed, small circular step, measuring 12.5 mm in diameter and having a thickness of 1.2 mm, was incorporated into the lower part of the slider. This step was designed to uniformly distribute the muscle force solely onto the sensing region of the FSR sensor. A small butyl rubber piece with a 1.2 mm thickness was attached to the circular step for a smooth, even force transfer onto the FSR sensor. The dimensions of the developed FSR sensor were 21 × 20 × 10 mm. Both the chassis and the slider of the casings were 3D-printed using polylactic acid (PLA) material. PLA is a rigid material with the required strength to sustain MC forces without failure. Figure 1 illustrates the step-by-step placement of the FSR sensor between both casings.

**Figure 2:**
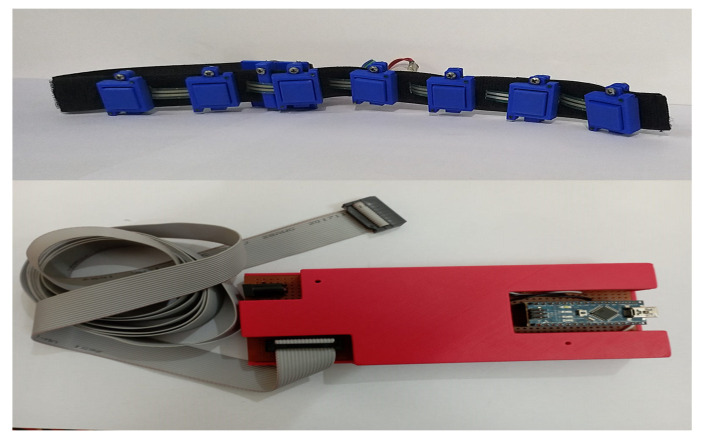
FMG band and signal conditioning circuitry.

Seven FSR sensor casings were attached to the Velcro strap’s 42 cm long loop part. The lower end of the casing contained the hook part of the Velcro strap, which made it easy to affix the casing firmly and helped change the sensor’s position on the strap. Figure 2 shows the FMG armband strap and signal conditioning circuit. The FMG band and casing position could be adjusted to fit various forearm circumferences.

#### 2.1.2. Signal Extraction and Conditioning Setup

**Figure 3:**
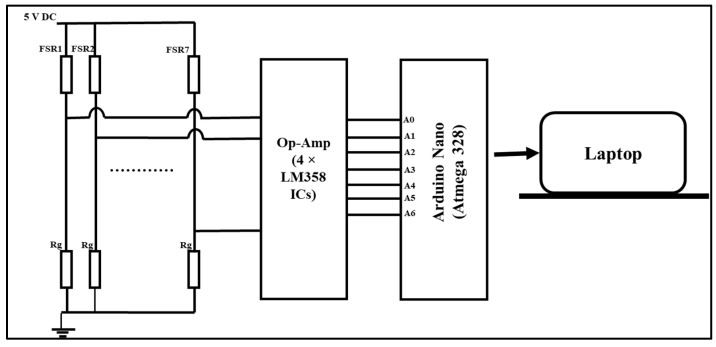
Schematic of FMG data acquisition.

An experimental setup consisting of the FMG band, data collection, and classification setup was developed. The FMG armband, with FMG sensors, measure volumetric changes in the muscles, and the FMG signals were digitized using an Arduino Nano board. The signals from the FSRs of the band were extracted using voltage divider circuit, as adopted in previous studies [49,50]. One of the two terminals of the FSR was connected to a 5 V DC supply, whereas the other terminal was connected to the voltage divider circuit. The voltage divider equation for the force-to-voltage conversion of the FSR sensor is shown below.

(1) $$V_{out}=\frac{R_{g\;}.V_{in}}{R_{g}+R_{FSR}}$$

where *R_g_* is the ground resistor (the value chosen in this study was 20 kΩ); *R_FSR_* is the resistance of the FSR sensor; and *V_in_* is the input voltage. The signals from the voltage divider circuit were digitized using an Arduino Nano board with a 16 MHz ATmega328 microprocessor and a 10-bit analog-to-digital converter (ADC). The schematic of the data acquisition from the FMG band is shown below in Figure 3.

### 2.2. Sensor Characterization

#### 2.2.1. Load vs. Voltage Characteristics

The sensitivity range of the newly developed FSR sensor was determined by placing different static loads from 0.1 kg to 1 kg on the sensing tip of the developed sensor. A load vs. output voltage curve was obtained, representing the developed sensor’s sensitivity range.

#### 2.2.2. Stability of FMG Signals

To determine the stability of FMG signals over time, the standard deviation (SD) of the FMG signals acquired during the execution of gestures was calculated. The stability over time of the signals was ascertained for single-hand, wrist, and forearm gestures, i.e., power, flexion, and supination.

### 2.3. Subjects

Six subjects (three healthy and three amputees) participated in this study. All the amputated subjects were trans-radial amputees and were presently using passive prostheses. The reason for recruiting only three amputee subjects was the difficulty of arranging and recruiting such subjects in this field for data collection [51,52]. The particulars of the subjects are shown in Table 1. All the subjects were physically active and did not have diabetes or skin-related disorders. The ethical committee of the University of Engineering and Technology Peshawar, Pakistan (UET, Peshawar), approved this study. All subjects were verbally and theoretically informed about the experimental procedure, and an informed consent form was duly signed before participating.

### 2.4. Protocol for Data Collection

**Figure 4:**
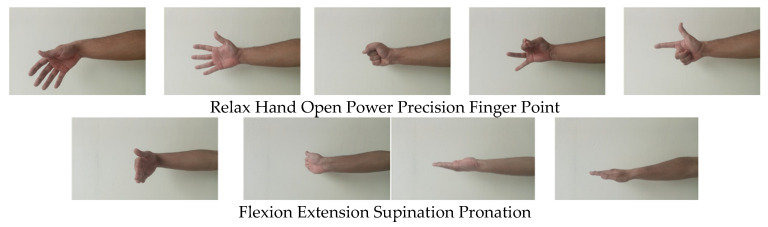
Selected gestures.

**Figure 5:**
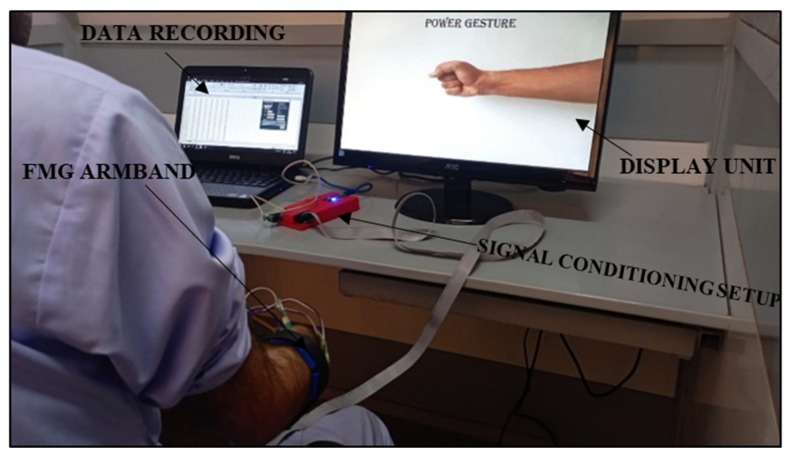
Experimental setup for data collection.

The FMG band was worn on the bulk area of the forearm to extract data. The first sensor was placed near the ulna bone, followed by the remaining sensors in an anti-clockwise direction for the right side of the forearm (clockwise for the left forearm). The band was fastened tight enough to ensure no slip or rotation during experimentation. None of the subjects complained about the tightness of the band. Data were collected for nine major upper limb gestures, which included 5 primary hand gestures [53], relax, hand open, power, precision (tripod), and finger point; two forearm gestures, supination and pronation [54]; and two wrist gestures [55], flexion and extension, as shown in Figure 4. During experimentation, the subjects were seated on a chair and asked to flex the experimental arm’s elbow at about 90°. For the convenience of the subjects, visual instructions about the gestures (gesture image along with its name) appeared on the display unit, and the subjects were asked to execute or mimic dispalyed gestures as shwn in Figure 5. Each gesture was held for five seconds, followed by a three-second relax gesture to avoid muscle fatigue during experimentation. The subjects were asked to repeat these gestures with repeatable and minimal force. The data were collected using the Parallax data acquisition tool (PLX-DAQ) [56]. Three trials were performed to acquire data from the developed band. A sampling frequency of 10 Hz was selected for the recording of data based on the Nyquist criteria for human hand motion (<4.5 Hz) [49]. Each trial lasted approximately 90 s, with a 15 min break provided to each subject between trials. During the experimentation, the subjects were free to rest or take breaks whenever desired. Following each trial, the FMG band was donned and removed from the subject’s forearm. During the initial two trials, the experimenter donned the armband on the subject’s forearm. However, in the final trial, the subjects donned the armband themselves and recorded data for the specified gestures.

Two experimental sessions were conducted, and data were recorded during both sessions. The procedures for band mounting and data collection remained consistent throughout. The only variation between the sessions was the arrangement of sensors on the FMG strap. In arrangement A, a fixed spacing distance (as adopted in previous studies [39,49]) of 4.5 cm between sensors was selected because this spacing distance was sufficient for all seven sensors to be equally distributed among the least thick forearms (subjects No. 2 and 6) available in this study. For arrangement B, instead of a fixed spacing between sensors, a uniform sensor distribution (as proposed in [2,37]) in which the sensors were equally spaced around the circumference of the forearm was adopted. Equation (2) was used for the equal distribution of sensors on the subject’s forearm.

(2) S=((R+T)×2π)) / N

**Figure 6:**
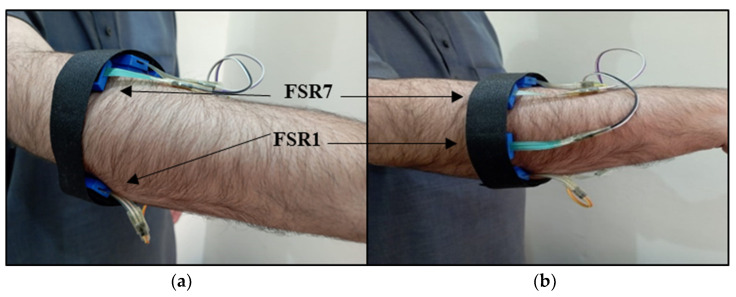
FMG armband: (**a**) arrangement A and (**b**) arrangement B configuration on subject No. 3.

Here, S is the spacing between sensors on the band, R is the radius of the forearm (bulk area of the forearm), T is the thickness of the FSR casing (1.2 cm), and N is the number of sensors. The distance between the sensors using Equation (2) is shown in Table 1. Figure 6 shows both sensor arrangements of the FMG band mounted on the subject’s forearm.

### 2.5. Data Collection and Analysis

The data collected from Section 2.4 were processed offline using MATLAB from MathWorks. The raw data without any feature extraction were used to classify gestures. The data sets were normalized between 0 and 1 using the maximum and minimum values in the concerned data sets. The classification of nine gestures was performed using commonly employed machine learning classifiers in the field of bio-signals, including support vector machines (SVMs) [50] with a Gaussian kernel and k-nearest neighbour [57]. A K-fold cross-validation scheme [42,58,59] with K = 5 was used. In this approach, the data were randomized and divided into five segments. Four segments were utilized for training, while the remainder of the data were allocated for testing. This process was repeated five times, ensuring all the data were used for training and testing. The average classification accuracies obtained from these five repetitions are reported in this paper.

## 3. Results

### 3.1. Sensor Characterization Results

#### 3.1.1. Load vs. Voltage

**Figure 7:**
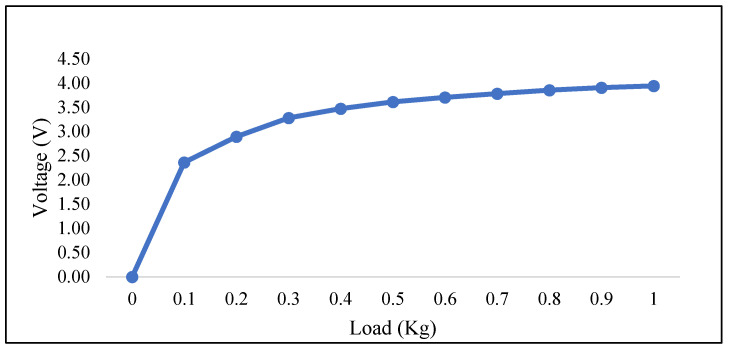
Force vs. voltage graph.

Figure 7 shows the load vs. voltage curve of the developed sensor for various loads. The curve slope shows the sensor’s non-linear relationship to the input load. The sensor’s measurement range for 0 to 1 kg of the load was determined to be 0 to 3.94 V.

#### 3.1.2. Stability of FMG Signals

**Figure 8:**
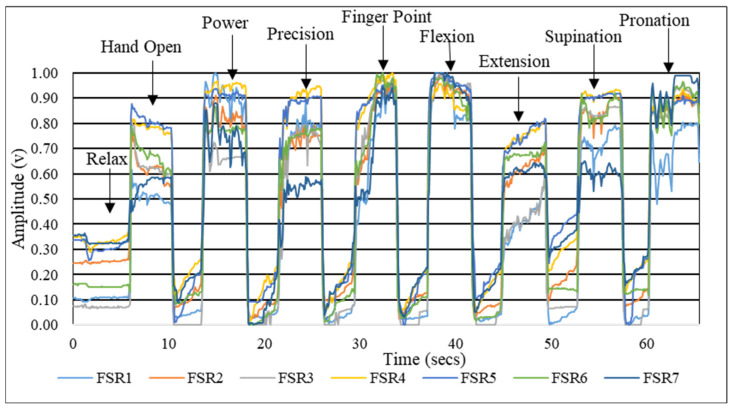
FMG signals acquired for various gestures.

**Figure 9:**
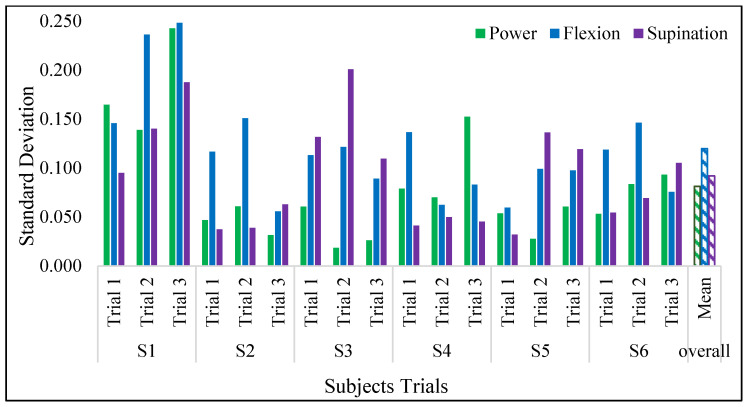
SD of the three gestures across the trials in subjects.

Figure 8 shows the FMG signals acquired during the execution of nine instructed gestures in a single trial. The signals corresponding to gestures are marked and separated by the 3 secs of the relax gesture. The SD of the FMG signals, obtained for all the subjects while executing gestures across the three trials, is shown in Figure 9. The minimum deviation was observed in the power gesture, whereas the maximum was in the flexion gesture. The mean deviation of the FMG signals across all subjects was 0.097.

### 3.2. Classification Comparison between Arrangement A and Arrangement B

The average classification accuracies for the overall cumulative subjects, hand gestures, wrist and forearm gestures, and individual subject inter-trials are presented in this section. Figure 13 shows the classification results for both sensor arrangements utilizing SVM and KNN classifiers.

#### 3.2.1. Overall Cumulative Classification Results

**Figure 10:**
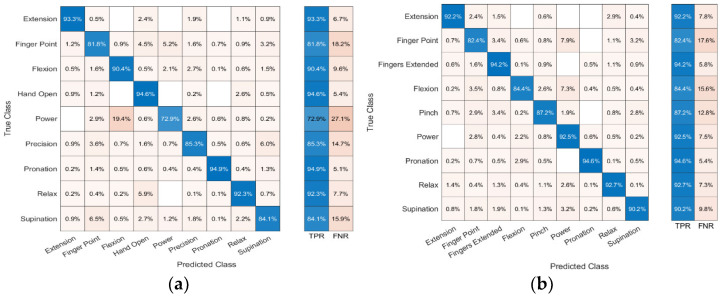
Confusion matrix of (**a**) arrangement A and (**b**) arrangement B using SVM.

**Figure 11:**
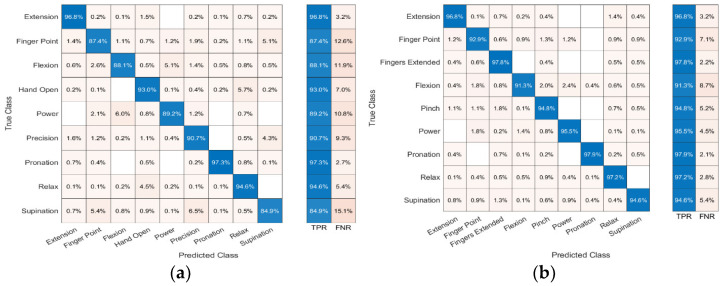
Confusion matrix of (**a**) arrangement A and (**b**) arrangement B using KNN.

The confusion matrices for predicting nine gestures across all six subjects are shown in Figure 10 and Figure 11. Figure 10 shows the classification accuracy for arrangement A, and Figure 11 shows the classification accuracy for arrangement B. In each confusion matrix, the rows and columns show the predicted and actual gestures, respectively. The entries along the diagonal, known as the true positive rate (TPR), depict the proportions of accurately classified gestures, while the entries off the diagonal signify the proportions of erroneously classified gestures. From the SVM, the average accuracies for arrangement A and arrangement B were 87.7 ± 3.3 and 90.0 ± 3.7%, respectively. The KNN classifier’s average accuracies for arrangement A and arrangement B were 91.3 ± 2.3% and 95.4 ± 2.1%, respectively. This demonstrates that arrangement B was slightly better at predicting gestures than arrangement A. Moreover, KNN performed better at predicting nine gestures for both sensor arrangements.

#### 3.2.2. Individual Subject Inter-Trial Classification Results

**Figure 12:**
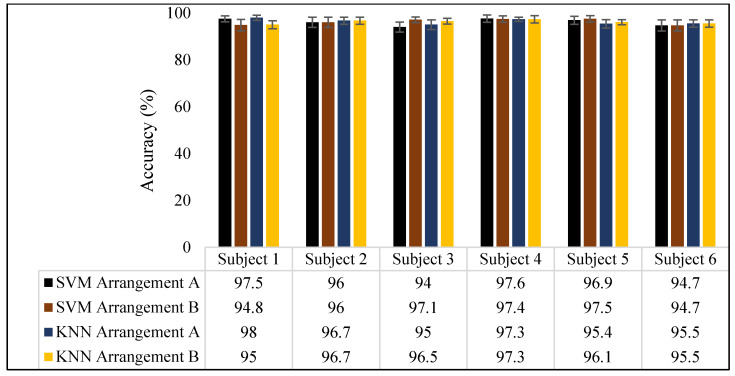
Individual subject classification results.

The average accuracy across the three trials of the individual subjects, acquired using both sensor arrangements, is presented in Figure 12. An average accuracy of obove 90% was observed for both sensor arrangements. Among the subjects, subject 4 showed a higher accuracy for the arrangement A configuration. For arrangement B, the maximum accuracy was found for subject 5.

#### 3.2.3. Hand Gesture Classification Results

**Figure 13:**
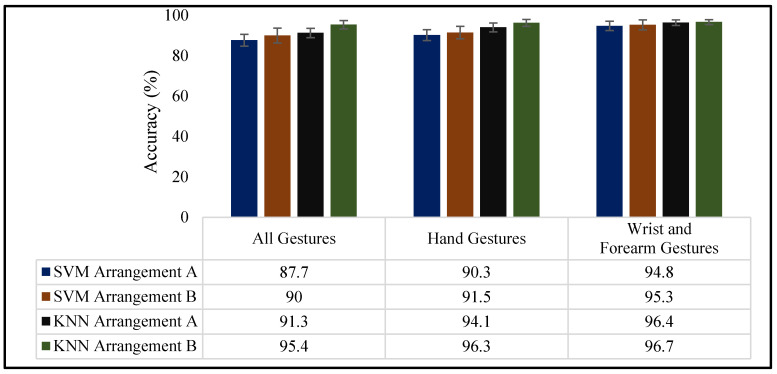
Average classification accuracies using arrangement A and arrangement B.

Figure 13 shows the average classification accuracies of the hand gestures (also known as fine-finger movements) obtained using the arrangement A and B bands. Both sensor arrangements achieved an average accuracy exceeding 90%. Specifically, with the SVM, average accuracies of 90.3 ± 3.1% and 91.5 ± 2.7% were attained for arrangements A and B, respectively. When employing KNN, average accuracies of 94.1 ± 2.2% and 96.2 ± 1.7% were recorded for arrangements A and B, respectively.

#### 3.2.4. Wrist and Forearm Gesture Classification Results

The wrist and forearm gestures displayed a superior accuracy compared to the hand gestures and all nine other categories. The average prediction accuracies, using both the SVM and KNN for arrangement A, were 94.7 ± 2.1% and 95.3 ± 1.%, respectively. In the case of arrangement B, the average prediction accuracy reached 96.4 ± 1.4% for the SVM and 96.7 ± 1.2% for KNN.

#### 3.2.5. Performance Evaluation and Comparative Analysis of Multiple Classifiers

**Figure 14:**
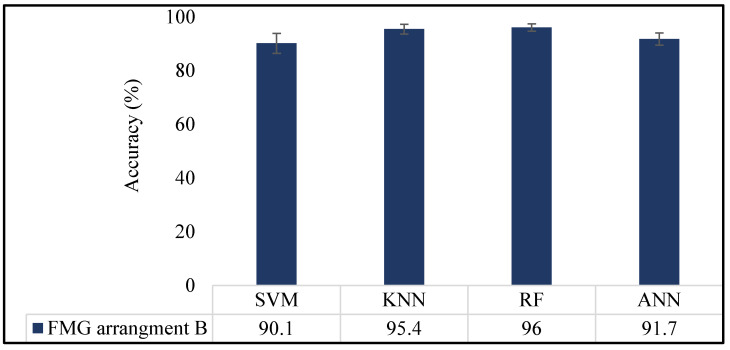
Four classifiers’ average classification accuracy.

The performance of the FMG band with sensor arrangement B was additionally assessed using classifiers, including random forest (RF) and artificial neural networks (ANNs). Figure 14 shows classification performance of multiple classifiers. Among these classifiers, RF stood out as the most effective in recognizing gestures compared to others. ANNs, on the other hand, achieved an average accuracy of 91.7 ± 2.3%. Notably, the SVM classifier demonstrated the lowest accuracy among these classifiers. This discrepancy might be attributed to SVM’s inherent nature as a binary classifier, as opposed to more robust classifiers such as ANNs and RF.

## 4. Discussion

An efficient gesture detection method is a fundamental requirement for the optimal control of bio-robotic devices. In this research, we introduced an FMG band, equipped with seven newly designed FMG sensors. As indicated by prior studies, this armband’s chosen number of sensors is believed to be sufficient for identifying grip patterns in control systems based on pattern recognition [35,39]. The decision regarding the number of sensors depends on the band’s stiffness, as each sensor contributes to the overall rigidity of the band [50]. Consequently, exceeding seven sensors increases the band’s stiffness, making it challenging to fit individuals with slender forearms.

### 4.1. Developed Sensor Characterization

The sensitivity of the developed sensors was not linear with respect to increasing loads. They were more sensitive to lighter loads as compared with heavier loads. This is believed to be beneficial in distinguishing different muscle pressure levels applied during grip patterns [48]. Moreover, in applications such as gesture recognition, the linearity of signals is less favorable, particularly when the signal patterns vary among different gestures. Another crucial attribute is the temporal stability of FSR signals. In this study, the FMG signals consistently exhibited a good stability (mean < 0.1). Power gestures in FMG signals demonstrated a relatively low variation compared to wrist and forearm gestures. A visual inspection of the FMG signal graphs also revealed that the FSR signals exhibited a greater stability in hand gestures than wrist and forearm gestures. The reason for this reduced variability in hand signals is that they primarily involved finger movements and could be executed without significantly affecting the elbow and shoulder position, unlike wrist and forearm gestures.

### 4.2. Classification Performance

The overall classification results suggested that the performance of arrangement B in predicting the nine gestures was slightly superior to that of arrangement A. An increase in the average accuracies around 2.5% and 4.5% were evident for the SVM and KNN, respectively, when using the arrangement B configuration for the FMG band. This increase in the prediction accuracy for arrangement B is likely attributable to the uniform distribution of sensors across the larger forearm area of all subjects, in contrast to arrangement A. In this study, subjects with varying forearm sizes were included to assess the FMG band’s comfort. It is worth noting that only two out of the six subjects (subjects No. 3 and 4) had a noticeable difference in sensor spacing, of approximately 0.5 cm, between the two sensor arrangements. These variations may explain the slight discrepancy in the prediction accuracies between the two sensor setups.

None of the sensor arrangement techniques exhibited consistent superiority across all subjects regarding the individual subject prediction performance. For specific subjects (such as subjects No. 3 and 5), arrangement B outperformed arrangement A in predicting gestures, while arrangement A showed dominance for others (such as subject No. 1). These accuracy variations were observed across the individual subjects during their three trials. Consequently, arrangement A demonstrated a superior performance for specific subjects, while arrangement B excelled for others.

The results for predicting hand gestures followed a similar trend, as observed in recognizing all nine gesture types. Arrangement A exhibited slightly lower performance, showing a 1.3% reduction in accuracy for SVM and a 2.3% reduction for KNN, compared to arrangement B when predicting hand gestures. However, both band configurations yielded nearly identical results in predicting wrist and forearm movements. The accuracy of predicting wrist and forearm gestures outperformed that of the hand gestures and all nine other gestures. Since wrist and forearm movements are gross-arm movements [33], each gesture in both sensor arrangements generated significantly distinguishable signal patterns compared to the gestures that involved finger movements.

Another noteworthy finding from this study is that the ratio between the number of sensors and the number of gestures influenced the accuracy in predicting gestures. When the number of sensors surpassed the number of gestures, the prediction accuracy tended to increase, and vice versa. This study predicted the wrist and forearm gestures (four in total) with a greater accuracy than all nine gestures, resulting in an approximate 8% accuracy improvement. This increase in accuracy due to an elevated sensor-to-gesture ratio aligned with the findings from previous research. Ahmadizedah et al. [33] demonstrated that the predictive accuracy could increase by as much as 11% by reducing the number of gesture categories from eight to three. Likewise, Jiang et al. [42] demonstrated that the accuracy can be improved by up to 4% by increasing the sensor quantity from 8 to 16 within the context of 52 gesture categories. Lei et al. [60] explored the relationship between the number of sensors and the number of gestures, revealing that the accuracy could be boosted by up to 24% when expanding the sensor count from 2 to 16.

### 4.3. FMG Band Wearability, Cost, Weight, and Satisfaction Survey

Regarding the comfort and ease of wearing the newly designed FMG band, it can be comfortably worn on the wrist, similar to a wristwatch, but it does require some support during attachment. The need for support during band attachment is primarily due to the size and weight of the casings designed for the FSRs, with each casing weighing approximately 12 g. The entire armband, including the sensors and wires, has a total weight of 97 g.

As of our knowledge cutoff date, there were no “medically certified” biocompatible FSR sensors available. In this study, the FSR sensors were enclosed in casings made of PLA material, which is known to be biocompatible and considered entirely safe for direct contact with human skin [61]. The cost of developing the FMG band and signal conditioning circuitry was approximately PKR 16,500 (equivalent to USD 93), with the FSR sensors priced at PKR 1050 each, the Arduino Nano at PKR 1650, and the wires at PKR 450. For reference, Table 2 compares our developed band with other research-based FMG bands.

The evaluation of the developed band and the experimental procedure to assess the band’s usability was conducted using the system usability scale (SUS) survey. This survey was exclusively administered to the trans-radial subjects (subjects No. 4–6), since they will ultimately serve as the end-users of the developed system. The SUS questionnaire, featuring questions rated on a 5-point scale ranging from “strongly disagree” to “strongly agree”, can be found in Appendix B. Table 3 presents the individual SUS scores for each subject, with the three subjects achieving an average score of 89.1. Subjects No. 4 and 6 initially encountered challenges when attempting the flexion and extension gestures. However, during the third data collection trial, all subjects managed to effortlessly and accurately wear the band.

### 4.4. Future Work

Future work should consider integrating these newly devised FMG sensors into trans-radial prostheses to create real-world end-user scenarios. These FMG sensors were designed with casing-based FSR sensors to be seamlessly accommodated within the prosthetic socket, similar to Ottobock 13E200 MyoBock EMG sensors. Additionally, it would be beneficial to expand the study by enlisting a broader range of trans-radial subjects, accounting for various anatomical shapes and sizes, and performing real-time gesture classification assessments. Various signal feature extraction methods, including time-domain, frequency-domain, and time–frequency-domain techniques, will be further investigated for the armband. The existing signal acquisition for the FMG band was conducted using a voltage divider circuit, demonstrating a non-linear response to an applied force. It is crucial to utilize a trans-impedance circuit to ensure that the signals are acquired in a linearly responsive manner to force [63]. Lastly, the developed band predicts dynamic upper limb gestures, since this study was conducted on fixed elbow and shoulder positions. The effect of elbow and shoulder movements needs to be considered.

## 5. Conclusions

In this research, a forearm armband employing force myography (FMG) technology was developed, equipped with a total of seven FMG sensors. These newly developed sensors were arranged into two distinct sensor configurations on the FMG band. The band was subjected to testing involving both individuals with intact limbs and amputees. Support vector machine (SVM) and k-nearest neighbor (KNN) algorithms were employed to predict upper limb gestures. The average accuracy results for arrangements A and B utilizing the SVM were 87.7 ± 3.3% and 90.0 ± 3.7%, respectively. Similarly, the average accuracies achieved with arrangement A and arrangement B utilizing the KNN classifier were 91.3 ± 2.3% and 95.4 ± 2.1%, respectively. Furthermore, the results indicated that enhancing the ratio of the quantity of sensors to the number of gestures led to an enhanced prediction accuracy. In this study, reducing the number of gestures to four led to around 8% increase in the prediction accuracy.

## Figures and Tables

**Table 1:** Particulars of subjects.

Subject ID No.	Healthy/Amputee	Gender	Age	Forearm Circumference (cm)	Arrangement B Configuration Sensor Distance (cm)	Experimentation Arm
1	Healthy	F	62	26	4.8	Left
2	Healthy	M	30	24	4.5	Right
3	Healthy	M	45	28	5.1	Right
4	Amputee	M	55	27	4.9	Right
5	Amputee	M	32	25	4.6	Right
6	Amputee	M	29	24	4.5	Right

**Table 2:** Comparison between research-based FMG bands.

FMG Band	No. of Sensors	Casing/Housing for FSRs	Sampling Frequency (Hz)	Weight (g)	Cost
Our developed band	7	Yes	10	97	USD 93
Ravindra et al. [34]	10	No	50	65	EUR 50
Menrva FMG band [39,49,50,62]	8	No	10–1k	-	-
Cannon et al. [2]	10	Yes	10	-	Below EUR 150
Jiang et al. [42]	16	No	10	-	USD 125
Ahmadizadeh et al. [33]	16	No	10	925, including socket weight	-
Ha et al. [38]	3	No	25	-	-

**Table 3:** SUS score of trans-radial subjects.

Subject ID No.	4	5	6	Mean
SUS score	82.5	95	90	89.11

**Figure A1:**
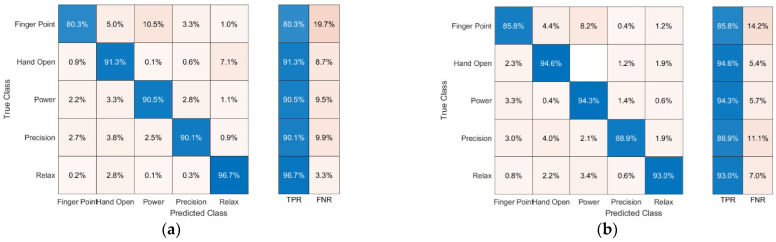
SVM confusion matrix for (**a**) arrangement A and (**b**) arrangement B while predicting hand gestures.

**Figure A2:**
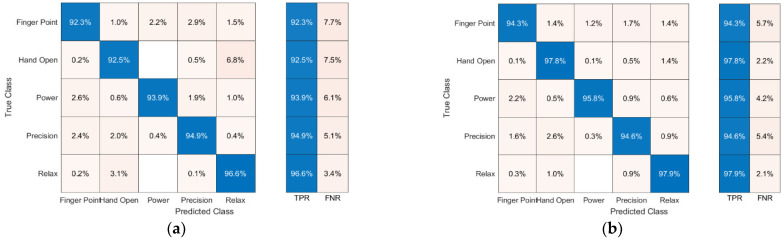
KNN confusion matrix for (**a**) arrangement A and (**b**) arrangement B while predicting hand gestures.

**Figure A3:**
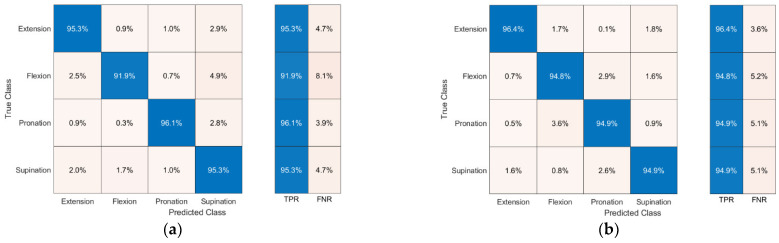
SVM confusion matrix for (**a**) arrangement A and (**b**) arrangement B while predicting wrist and forearm gestures.

**Figure A4:**
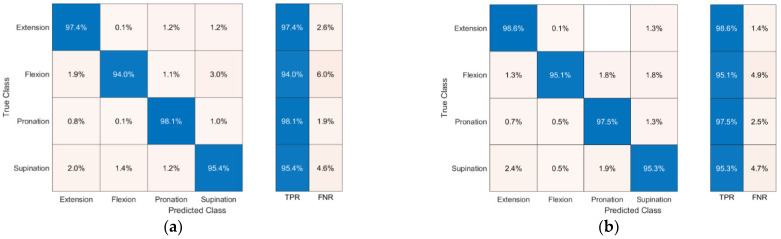
KNN confusion matrix for (**a**) arrangement A and (**b**) arrangement B while predicting wrist and forearm gestures.

## Data Availability

Data available on request due to restrictions.

## Appendix B

**SUS Survey:**

- I would like to use this band frequently.
- I found the band unnecessarily complex.
- I thought the band was easy to use.
- I think that I would need the support of a technical person to be able to use this system.
- I found the various functions in this experimental setup were well-integrated.
- I thought there was too much inconsistency in this band.
- I would imagine that most people would learn how to use this band very quickly.
- I found the band very cumbersome to use.
- I felt very confident using the band.
- I needed to learn a lot of things before I could get going with the experimental setup.

## References

[1] Sahu A., Sagar R., Sarkar S., Sagar S. (2016). Psychological effects of amputation: A review of studies from India. Ind. Psychiatry J..

[2] Connan M., Ruiz Ramírez E., Vodermayer B., Castellini C. (2016). Assessment of a wearable force-and electromyography device and comparison of the related signals for myocontrol. Front. Neurorobot..

[3] Sellegren K.R. (1982). An early history of lower limb amputations and prostheses. Iowa Orthop. J..

[4] Vitali M. (1978). Amputations and Prostheses.

[5] Kerr M., Barron E., Chadwick P., Evans T., Kong W., Rayman G., Sutton-Smith M., Todd G., Young B., Jeffcoate W.J. (2019). The cost of diabetic foot ulcers and amputations to the National Health Service in England. Diabet. Med..

[6] National Servies Scotland (2014). Number of Upper and Lower Limb Amputations Performed Each Year by the NHS in Scotland from 1981 to 2013. https://nhsnss.org/media/1397.

[7] Ziegler-Graham K., MacKenzie E.J., Ephraim P.L., Travison T.G., Brookmeyer R. (2008). Estimating the prevalence of limb loss in the United States: 2005 to 2050. Arch. Phys. Med. Rehabil..

[8] Semasinghe C., Prasanna J., Kandamby H., Ranaweera R., Madusanka D., Gopura R. Transradial Prostheses: Current Status and Future Directions. Proceedings of the 2016 Manufacturing & Industrial Engineering Symposium (MIES).

[9] Biddiss E.A., Chau T.T. (2007). Upper limb prosthesis use and abandonment: A survey of the last 25 years. Prosthet. Orthot. Int..

[10] Geethanjali P., Ray K., Shanmuganathan P.V. Actuation of prosthetic drive using EMG signal. Proceedings of the TENCON 2009–2009 IEEE Region 10 Conference.

[11] Merletti R., Parker P.J. (2004). Electromyography: Physiology, Engineering, and Non-Invasive Applications.

[12] Scott R.N., Parker P.A. (1988). Myoelectric prostheses: State of the art. J. Med Eng. Technol..

[13] Kamavuako E.N., Rosenvang J.C., Bøg M.F., Smidstrup A., Erkocevic E., Niemeier M.J., Jensen W., Farina D. (2013). Influence of the feature space on the estimation of hand grasping force from intramuscular EMG. Biomed. Signal Process. Control..

[14] Hargrove L.J., Englehart K., Hudgins B. (2007). A comparison of surface and intramuscular myoelectric signal classification. IEEE Trans. Biomed. Eng..

[15] Oskoei M.A., Hu H. (2007). Myoelectric control systems—A survey. Biomed. Signal Process. Control..

[16] Criswell E. (2010). Cram’s Introduction to Surface Electromyography.

[17] Merletti R., Aventaggiato M., Botter A., Holobar A., Marateb H., Vieira T. (2010). Advances in surface EMG: Recent progress in detection and processing techniques. Crit. Rev. Biomed. Eng..

[18] Cavanagh P.R., Komi P.V. (1979). Electromechanical delay in human skeletal muscle under concentric and eccentric contractions. Eur. J. Appl. Physiol..

[19] Viitasalo J.T., Komi P.V. (1981). Interrelationships between electromyographic, mechanical, muscle structure and reflex time measurements in man. Acta Physiol. Scand..

[20] Esposito D., Gargiulo G.D., Parajuli N., Cesarelli G., Andreozzi E., Bifulco P. Measurement of muscle contraction timing for prosthesis control: A comparison between electromyography and force-myography. Proceedings of the 2020 IEEE International Symposium on Medical Measurements and Applications (MeMeA).

[21] Esposito F., Limonta E., Cè E. (2011). Passive stretching effects on electromechanical delay and time course of recovery in human skeletal muscle: New insights from an electromyographic and mechanomyographic combined approach. Eur. J. Appl. Physiol..

[22] Fougner A., Scheme E., Chan A.D., Englehart K., Stavdahl Ø. (2011). Resolving the limb position effect in myoelectric pattern recognition. IEEE Trans. Neural Syst. Rehabil. Eng..

[23] Jiang N., Dosen S., Muller K.-R., Farina D. (2012). Myoelectric control of artificial limbs—Is there a need to change focus? [In the spotlight]. IEEE Signal Process. Mag..

[24] Sikdar S., Rangwala H., Eastlake E.B., Hunt I.A., Nelson A.J., Devanathan J., Shin A., Pancrazio J.J. (2013). Novel method for predicting dexterous individual finger movements by imaging muscle activity using a wearable ultrasonic system. IEEE Trans. Neural Syst. Rehabil. Eng..

[25] Esposito D., Andreozzi E., Fratini A., Gargiulo G.D., Savino S., Niola V., Bifulco P. (2018). A piezoresistive sensor to measure muscle contraction and mechanomyography. Sensors.

[26] Sturma A., Stamm T., Hruby L.A., Bischof B., Salminger S., Gstoettner C., Prahm C., Pittermann A., Wakolbinger R., Hofer C. (2022). Rehabilitation of high upper limb amputees after Targeted Muscle Reinnervation. J. Hand Ther..

[27] Xiao Z.G., Menon C., Menon C. (2019). A review of force myography research and development. Sensors.

[28] Prakash A., Sahi A.K., Sharma N., Sharma S. (2020). Force myography controlled multifunctional hand prosthesis for upper-limb amputees. Biomed. Signal Process. Control.

[29] Ahmadizadeh C., Pousett B., Menon C. (2019). Investigation of channel selection for gesture classification for prosthesis control using force myography: A case study. Front. Bioeng. Biotechnol..

[30] Esposito D., Savino S., Andreozzi E., Cosenza C., Niola V., Bifulco P. (2021). The “Federica” Hand. Bioengineering.

[31] Booth R., Goldsmith P. (2018). A wrist-worn piezoelectric sensor array for gesture input. J. Med Biol. Eng..

[32] Yungher D., Craelius W. Discriminating 6 grasps using force myography of the forearm. Proceedings of the American Society of Biomechanics Northeast Conference.

[33] Ahmadizadeh C., Merhi L.-K., Pousett B., Sangha S., Menon C. (2017). Toward intuitive prosthetic control: Solving common issues using force myography, surface electromyography, and pattern recognition in a pilot case study. IEEE Robot. Autom. Mag..

[34] Ravindra V., Castellini C. (2014). A comparative analysis of three non-invasive human-machine interfaces for the disabled. Front. Neurorobotics.

[35] Wininger M., Kim N.-H., Craelius W. (2008). Pressure signature of forearm as predictor of grip force. J. Rehabil. Res. Dev..

[36] Li N., Yang D., Jiang L., Liu H., Cai H. (2012). Combined use of FSR sensor array and SVM classifier for finger motion recognition based on pressure distribution map. J. Bionic Eng..

[37] Xiao Z.G., Menon C. (2017). Performance of forearm FMG and sEMG for estimating elbow, forearm and wrist positions. J. Bionic Eng..

[38] Ha N., Withanachchi G.P., Yihun Y. (2019). Performance of forearm FMG for estimating hand gestures and prosthetic hand control. J. Bionic Eng..

[39] Xiao Z.G., Menon C. (2014). Towards the development of a wearable feedback system for monitoring the activities of the upper-extremities. J. Neuroeng. Rehabil..

[40] Radmand A., Scheme E., Englehart K. High-resolution muscle pressure mapping for upper-limb prosthetic control. Proceedings of the MEC–Myoelectric Control Symposium.

[41] Castellini C., Kõiva R., Pasluosta C., Viegas C., Eskofier B. (2018). Tactile myography: An off-line assessment of able-bodied subjects and one upper-limb amputee. Technologies.

[42] Jiang X., Merhi L.-K., Xiao Z.G., Menon C. (2017). Exploration of force myography and surface electromyography in hand gesture classification. Med. Eng. Phys..

[43] Chapman J., Dwivedi A., Liarokapis M. A wearable, open-source, lightweight forcemyography armband: On intuitive, robust muscle-machine interfaces. Proceedings of the 2021 IEEE/RSJ International Conference on Intelligent Robots and Systems (IROS).

[44] Anvaripour M., Khoshnam M., Menon C., Saif M. (2020). FMG-and RNN-based estimation of motor intention of upper-limb motion in human-robot collaboration. Front. Robot. AI.

[45] Xiao Z.G., Menon C. (2017). Counting grasping action using force myography: An exploratory study with healthy individuals. JMIR Rehabil. Assist. Technol..

[46] Rehman M.U., Shah K., Haq I.U., Khurshid H. A Force Myography based HMI for Classification of Upper Extremity Gestures. Proceedings of the 2022 2nd International Conference on Artificial Intelligence (ICAI).

[47] Rehman M.U., Shah K., Haq I.U., Iqbal S., Ismail M.A., Selimefendigil F. (2023). Assessment of Low-Density Force Myography Armband for Classification of Upper Limb Gestures. Sensors.

[48] Prakash A., Sharma N., Sharma S. (2020). Novel force myography sensor to measure muscle contractions for controlling hand prostheses. Instrum. Sci. Technol..

[49] Cho E., Chen R., Merhi L.-K., Xiao Z., Pousett B., Menon C. (2016). Force myography to control robotic upper extremity prostheses: A feasibility study. Front. Bioeng. Biotechnol..

[50] Sadarangani G.P., Jiang X., Simpson L.A., Eng J.J., Menon C. (2017). Force myography for monitoring grasping in individuals with stroke with mild to moderate upper-extremity impairments: A preliminary investigation in a controlled environment. Front. Bioeng. Biotechnol..

[51] Atzori M., Gijsberts A., Castellini C., Caputo B., Hager A.-G.M., Elsig S., Giatsidis G., Bassetto F., Müller H. (2014). Electromyography data for non-invasive naturally-controlled robotic hand prostheses. Sci. Data.

[52] Atzori M., Gijsberts A., Müller H., Caputo B. Classification of hand movements in amputated subjects by sEMG and accelerometers. Proceedings of the 2014 36th Annual International Conference of the IEEE Engineering in Medicine and Biology Society.

[53] Hermens H., Stramigioli S., Rietman H., Veltink P., Misra S. (2011). Myoelectric forearm prostheses: State of the art from a user-centered perspective. J. Rehabil. Res. Dev..

[54] Mizuno H., Tsujiuchi N., Koizumi T. Forearm motion discrimination technique using real-time EMG signals. Proceedings of the 2011 Annual International Conference of the IEEE Engineering in Medicine and Biology Society.

[55] Sarrafian S.K., Melamed J.L., Goshgarian G. (1977). Study of wrist motion in flexion and extension. Clin. Orthop. Relat. Res..

[56] PLX-DAQ|Parallax. 26 June 2021. https://www.parallax.com/package/plx-daq/.

[57] Narayan Y. (2021). SEMG signal classification using KNN classifier with FD and TFD features. Mater. Today Proc..

[58] Ferigo D., Merhi L.-K., Pousett B., Xiao Z.G., Menon C. (2017). A case study of a force-myography controlled bionic hand mitigating limb position effect. J. Bionic Eng..

[59] Javaid H.A., Tiwana M.I., Alsanad A., Iqbal J., Riaz M.T., Ahmad S., Almisned F.A. (2021). Classification of Hand Movements Using MYO Armband on an Embedded Platform. Electronics.

[60] Lei G., Zhang S., Fang Y., Wang Y., Zhang X. (2021). Investigation on the Sampling Frequency and Channel Number for Force Myography Based Hand Gesture Recognition. Sensors.

[61] Ramot Y., Haim-Zada M., Domb A.J., Nyska A. (2016). Biocompatibility and safety of PLA and its copolymers. Adv. Drug Deliv. Rev..

[62] Xiao Z.G., Menon C. (2019). An investigation on the sampling frequency of the upper-limb force myographic signals. Sensors.

[63] Paredes-Madrid L., Palacio C.A., Matute A., Parra Vargas C.A. (2017). Underlying physics of conductive polymer composites and force sensing resistors (FSRs) under static loading conditions. Sensors.

